# Periodic Stratified Porous Structures in Dynamic Polyelectrolyte Films Through Standing‐Wave Optical Crosslinking for Structural Color

**DOI:** 10.1002/advs.202100402

**Published:** 2021-05-27

**Authors:** Wei‐Pin Huang, Hong‐Lin Qian, Jing Wang, Ke‐Feng Ren, Jian Ji

**Affiliations:** ^1^ MOE Key Laboratory of Macromolecule Synthesis and Functionalization Department of Polymer Science and Engineering Zhejiang University Hangzhou 310027 China; ^2^ Key Laboratory of Cardiovascular Intervention and Regenerative Medicine of Zhejiang Province Department of Cardiology Sir Run Run Shaw Hospital Zhejiang University Hangzhou 310016 China

**Keywords:** periodic structures, photo‐cross‐linkable polyelectrolytes, polyelectrolyte film, standing‐wave optics, structural color

## Abstract

Periodic porous structures have been introduced into functional films to meet the requirements of various applications. Though many approaches have been developed to generate desired structures in polymeric films, few of them can effectively and dynamically achieve periodic porous structures. Here, a facile way is proposed to introduce periodic stratified porous structures into polyelectrolyte films. A photo‐crosslinkable polyelectrolyte film of poly(ethylenimine) (PEI) and photoreactive poly(acrylic acid) derivative (PAA‐N_3_) is prepared by layer‐by‐layer (LbL) self‐assembly. Stratified crosslinking of the PEI/PAA‐N_3_ film is generated basing on standing‐wave optics. The periodic stratified porous structure is constructed by forming pores in noncrosslinked regions in the film. Thanks to the dynamic mobility of polyelectrolytes, this structural controlment can be repeated several times. The size of pores corresponding to the layer spacing of the film contributes to the structural colors. Furthermore, structural color patterns are fabricated in the film by selective photo‐crosslinking using photomasks. Although the large‐scale structural controlment in thick (micron‐scale and above) films needs to be explored further, this work highlights the periodic structural controlment in polymeric films and thus presents an approach for application potentials in sensor, detection, and ink‐free printing.

## Graphical abstract

The periodic stratified porous structures are generated in polyelectrolyte film through periodic crosslinking based on the standing‐wave optics. The film at solid‐state and porous‐state is switched reversibly. Subsequently, the film displays different structural color.

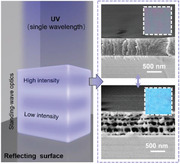

## 1 Introduction

Porous structural films are used in a wide range of applications for drug delivery, optoelectronics, catalysis, separation, where structural features are essential for diversities in material functionalization.^[^ ^1^ ^]^ For instance, tunable wettability could be obtained by adjusting the shapes of pores.^[^ ^2^ ^]^ Micrometer‐sized voids were utilized in the rapid transportation of reactive molecules in a battery.^[^ ^3^ ^]^ Especially, regulating the porous structures to be periodic and stratified is much more important for material functionalization, which can be vividly exhibited in biological systems. Wood possessing hierarchical porous structures presents flexibility and high strength;^[^ ^4^ ^]^ nacre with stratified organic–inorganic structure exhibits both optical iridescence and impact resistance;^[^ ^5^ ^]^ whale baleen consisting of a series of parallel plates and pores possesses a high fracture toughness.^[^ ^6^ ^]^ Inspired by these, polymeric films with periodic and stratified porous structures have been dreamed about by scientists and engineers for decades.

Polyelectrolyte films driven by electrostatic interaction can be typically fabricated from layer‐by‐layer (LbL) assembly.^[^ ^7^ ^]^ By adjusting the electrostatic interaction between polyelectrolytes or introducing other covalent/non‐covalent interactions, the film structures will be expected to be tuned delicately and dynamically.^[^ ^8^ ^]^ Recently, we presented a way to reversibly construct and eliminate porous structures in the polyelectrolyte film.^[^ ^9^ ^]^ The charged density of polyelectrolytes was influenced by an acid solution treatment, leading to the development of microphase separation and consequently the formation of micro‐/nano‐porous structures.^[9c]^ Furthermore, the mobility of polyelectrolyte can be enhanced by water plasticization, and then the porous structures were eliminated under relative saturated humidity (100% RH) environment.^[^ ^10^ ^]^ The procedures above were reversible. Very recently, we introduced covalent/non‐covalent interactions into polyelectrolyte films to construct pores regionally and sequentially.^[^ ^11^ ^]^ Nevertheless, the pore‐formation in these studies is random rather than periodic and regular.

**Scheme 1:**
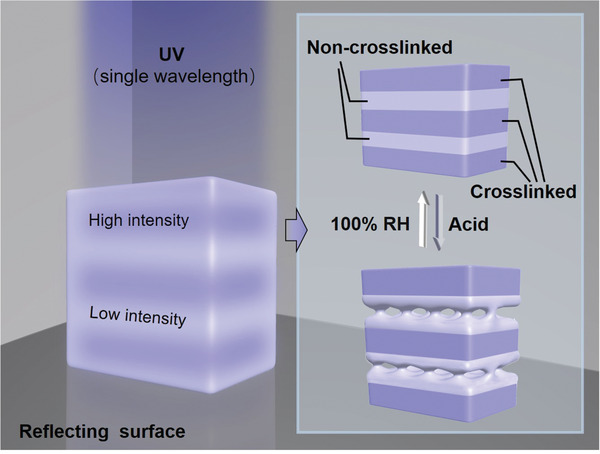
The schematic of the construction of periodic stratified porous structures in dynamic polyelectrolyte film. The stratified crosslinking of PEI/PAA‐N_3_ film under UV light with the standing‐wave feature. A beam of light oppositely encounters its reflected light and then the standing‐wave optics develops. The pore‐formation in non‐crosslinked layers was achieved in a bath of acid solution. The erasure of micropores was achieved under 100% RH environment.

Given that the pore formation was due to the mobility and consequent microphase separation of the free polyelectrolytes (non‐covalent bonded) in the film during the acid treatment as we previously reported,^[9c,10]^ we reasoned that the film with periodic porous structure might be achieved if we can selectively crosslink the polyelectrolytes at a periodic pattern. Sivaniah and co‐workers have recently reported that the multi‐layered crosslinking could be introduced into polymeric film by standing‐wave optics.^[^ ^12^ ^]^ Inspired by this, we proposed a modified way to prepare dynamic stratified porous structures in polyelectrolyte films by controlling the dynamics of polyelectrolytes (**Scheme** **1**). Cationic poly(ethylenimine) (PEI) and photoreactive anionic poly(acrylic acid) (PAA) derivative (PAA‐N_3_) were generated a photo‐reactive polyelectrolyte film (PEI/PAA‐N_3_). A beam of single‐wavelength light oppositely encounters its reflected light and then the standing‐wave optics develops, whose intensity has a periodic distribution. After being exposed to the standing‐wave optics, the PEI/PAA‐N_3_ film could be crosslinked hierarchically. Stratified porous structures can be created by immersing the film in a bath of acid solution. After that, the porous structures can be eliminated in 100% RH environment. Based on it, a polyelectrolyte film with tunable structural color was prepared. This dynamic strategy provides a new concept for controlling periodic structures of polymeric films and paving the way to explore new applications.

## 2 Results and Discussion

### 2.1 Fabrication of Photo‐Crosslinkable PEI/PAA‐N_3_ Film

**Figure 1:**
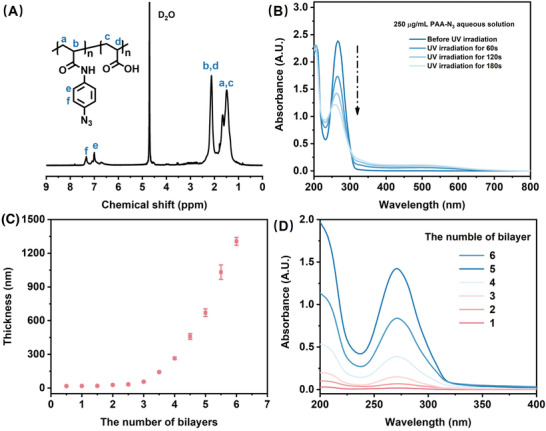
A) The ^1^H‐NMR spectrum of PAA‐N_3_. B) The UV–vis spectra of PAA‐N_3_ aqueous solution (250 µg mL^−1^) before and after UV irradiation for different time (60, 120, and 180 s). C) The thickness tendency of PEI/PAA‐N_3_ films with the increase of the number of bilayers. Data were represented as mean ± SD of *n* = 3 technical replicates. D) The UV–vis spectra of PEI/PAA‐N_3_ films with the different number of bilayers.

In this study, developing a cross‐linkable polyelectrolyte film is the foremost step to the formation of periodic stratified porous structures. It had been reported that azido groups had a broad absorbance at 250 to 300 nm, which could be typically activated to generate covalent bonds with polymeric backbone upon 365 nm light irradiation.^[^ ^13^ ^]^ Hence, we intended to introduce azido groups into the PEI/PAA film to obtain a photo‐reactive polyelectrolyte film. We firstly grafted phenyl azido moieties onto PAA by amidation reaction.^[^ ^13^, ^14^ ^]^ Proton nuclear magnetic resonance (^1^H‐NMR) was utilized to verify the success of the grafting reaction. As shown in **Figure** **1A**, absorption peaks of 6.8–7.8 ppm appeared in ^1^H‐NMR spectroscopy, and the calculated grafting ratio was 5.5%. It indicated the expected PAA‐N_3_ was synthesized successfully. To demonstrate the photo‐reactivity of PAA‐N_3_, we measured the absorption spectra of PAA‐N_3_ aqueous solution before and after UV irradiation (Figure 1B). The absorbance peak at 265 nm corresponding to *π*–*π* conjugation between benzene and azido moieties decreased obviously, illustrating the favorable photo‐response of PAA‐N_3_ to 365 nm light.^[^ ^13^, ^15^ ^]^

After that, polyelectrolyte films were generated through alternate deposition of cationic PEI and anionic derivative PAA‐N_3_. To trace the procedure of LbL assembly, the thickness with the increase of layers was characterized. As shown in Figure 1C, the PEI/PAA‐N_3_ films showed an exponential growth mode at the beginning and then switched into a linear growth mode. The former mode was attributed to diffusion “in” and “out” of polyelectrolytes during deposition,^[^ ^16^ ^]^ and the latter one mainly depended on the inadequate deposition time (15 min in this case) for the subsequent assembly.^[^ ^17^ ^]^ It conformed to the LbL self‐assembly behavior, proving the preparation of polyelectrolyte films. We also monitored the absorbance spectra with the increase of layers. In Figure 1D, the absorption peak of the azido groups was at 271 nm. As the number of layers increases, the absorbance peaks enhanced gradually, which indicated that we had accomplished introducing azido groups into PEI/PAA‐N_3_ films. For the following‐up experiments, we chose the (PEI/PAA‐N_3_)_5_ film with a 670.7 ± 33.7 nm thickness as samples. Herein, the film refers to (PEI/PAA‐N_3_)*_n_*, where *n* means the number of bilayers.

### 2.2 Stratified Crosslinking of the (PEI/PAA‐N_3_)_5_ Film

**Figure 2:**
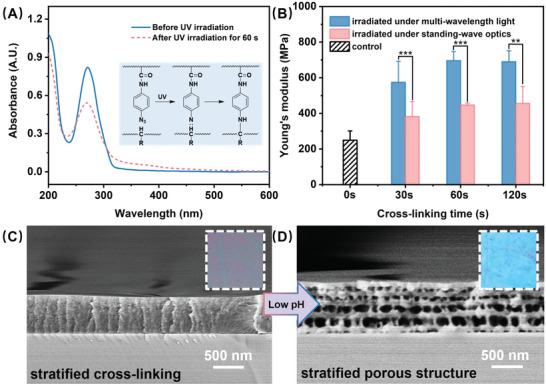
A) The absorption spectra of the (PEI/PAA‐N_3_)_5_ film before and after UV irradiation (a 365 nm single‐wavelength light, 60 s). The (PEI/PAA‐N_3_)_5_ film assembled on a quartz plate was placed onto a silicon wafer to apply a reflected surface during UV irradiation. The insert is the schematic of the photo‐crosslinking chemistry. B) The Young's modulus of (PEI/PAA‐N_3_)_5_ films after irradiation with single‐wavelength light and multi‐wavelength light for various time (0, 30, 60, and 120 s). Data were represented as mean ± SD of *n* = 5 technical replicates. Significance was considered as *p < 0.05, **p < 0.01, and ***p < 0.001. C) The cross‐sectional SEM image of the (PEI/PAA‐N_3_)_5_ films illuminated in single‐wavelength light (365 nm, 60 s). The insert refers to the visual color of the stratified crosslinked (PEI/PAA‐N_3_)_5_ films. D) The cross‐sectional SEM image of the stratified crosslinked (PEI/PAA‐N_3_)_5_ films after acid treatment (HCl aqueous solution, pH 2.3, 6 min). The insert refers to the visual color of the stratified porous (PEI/PAA‐N_3_)_5_ films.

The photo‐reactive property of (PEI/PAA‐N_3_)_5_ films is crucial. To further testify it, we exposed the (PEI/PAA‐N_3_)_5_ film on a reflected surface into the irradiation from a beam of single‐wavelength light (365 nm) for 60 s. The absorbance would be decreased if the photo‐reaction of azido groups was activated.^[9b,13]^ Thus its absorption spectrum was measured and analyzed (**Figure** **2A**). The absorbance showed a 34.1% reduction (from 0.82 to 0.54) after irradiation. The photo‐reaction induced covalent bonds into the (PEI/PAA‐N_3_)_5_ film (as shown in the insert in Figure 2A), which should make an impact on the film mechanical properties. As shown in Figure 2B, after crosslinking in the standing‐wave optics,^[12a]^ Young's modulus had an increase from 256.8 ± 69.3 to 456.2 ± 96.4 MPa. In contrast, the film upon multi‐wavelength light irradiation for 120 s showed 690.1 ± 60.8 MPa. Such a difference in the stiffness may be ascribed to different crosslinking densities since the multi‐wavelength light leads to whole film crosslinking while the standing‐wave light leads to part of the film.^[^ ^18^ ^]^

### 2.3 Construction of Periodic Stratified Porous Structure

The (PEI/PAA‐N_3_)_5_ film has a very dynamic property in physical structure as we previously demonstrated. ^[9c,10]^ We immersed the as‐prepared (PEI/PAA‐N_3_)_5_ film in a bath of HCl solution (pH 2.3) for 6 min and got random pores in the film, as shown in Figure S1A,B (Supporting Information). After that, the porous film was switch to a flat and solid‐state at 100% RH environment (Figure S1C, Supporting Information) due to the plasticization and electrostatic shielding of water molecules.^[^ ^19^ ^]^ In contrast, after being irradiated under multi‐wavelength light, the polyelectrolytes in the (PEI/PAA‐N_3_)_5_ film were restrained. Thus, the film kept a solid‐state during the acid treatment, as shown in Figure S2A,B (Supporting Information). To achieve periodic stratified porous structure, we irradiated the (PEI/PAA‐N_3_)_5_ film using standing‐wave optics for 60 s (Figure 2C) and then dipped it into HCl aqueous solution (pH 2.3) for 6 min. One can observe the stratified pores in the film with ≈100 nm layer spacing (Figure 2D). It was reasonable to speculate that the existence of solid layers was ascribed to the formation of covalent bonds and the porous layers corresponded to the non‐crosslinked regions.^[14a]^ In this way, we hierarchically controlled the mobility of polyelectrolytes in the photo‐reactive (PEI/PAA‐N_3_)_5_ film and thus achieved the periodic stratified porous structure. The same structure could be generated in a (PEI/PAA‐N_3_)_6_ film based on the strategy (Figure S3, Supporting Information). While the low UV light transmission and poor quality of the standing‐wave optics would limit the formation of periodic stratified porous in a thicker film.

Since the delicate stratified pores were prepared, the (PEI/PAA‐N_3_)_5_ film displayed a bright blue color rather than the gray color of the solid film, as shown in the inserts in Figure 2C,D. It is known that the structural color could be developed by optical diffraction, interference, dispersion, and scattering.^[^ ^20^ ^]^ Nevertheless, stratified structures generally lead to structural color through optical interference (Figure S4, Supporting Information).^[^ ^21^ ^]^ After pore‐formation, the refractive indices in crosslinked and non‐crosslinked layers were different,^[^ ^22^ ^]^ and then the visible light can be reflected in these interfaces.

### 2.4 Tunability of Periodic Stratified Porous Structure of Dynamic PEI/PAA‐N_3_ Film

**Figure 3:**
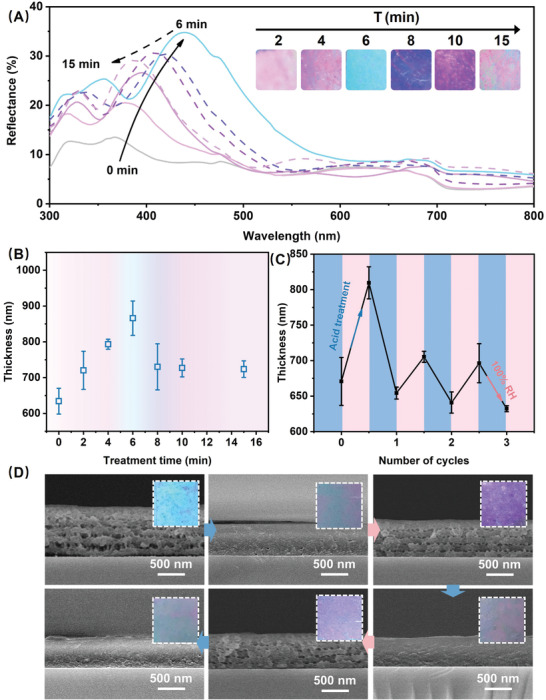
A) The reflectance spectra of (PEI/PAA‐N_3_)_5_ films after acid treatment (HCl aqueous solution, pH 2.3) for different time (0, 2, 4, 6, 8, 10, and 15 min). The inserts stand for the visual color of the films after acid treatment (HCl aqueous solution, pH 2.3) for different time (2, 4, 6, 8, 10, and 15 min). B) The thickness of (PEI/PAA‐N_3_)_5_ films after acid treatment (HCl aqueous solution, pH 2.3) for different time (0, 2, 4, 6, 8, 10, and 15 min). Data were represented as mean ± SD of *n* = 3 technical replicates. C) The thickness change of (PEI/PAA‐N_3_)_5_ films after different circulations of acid treatment for 6 min and exposure into 100% RH environment for 12 h. Data were represented as mean ± SD of *n* = 3 technical replicates. D) The recycles of the preparation of stratified porous structures and the erasure of pores in a (PEI/PAA‐N_3_)_5_ film. The inserts were the visual color of the film in different stages.

The gap spacing between two solid layers could significantly influence the structural color.^[^ ^23^ ^]^ In this study, we used the UV light with a fixed wavelength so that the crosslinked layers had a fixed thickness. Combining with the dynamic property of polyelectrolyte films, we attempted to adjust the thickness of the porous layer to tune the structural color. Through immersing the stratified crosslinked (PEI/PAA‐N_3_)_5_ film into the acid solution for various time, we found that the films displayed different colors. **Figure** **3A** showed reflectance spectra of these films. The reflectance of the films increased with the acid treatment time up to 6 min, showing a red shift. While longer treatment of the film led to a decrease of the reflectance along with the blue shift of reflection peaks. It is reported that a decrease of the layer spacing would generate a blue shift of the spectrum.^[^ ^24^ ^]^ Based on these, we assumed that during the process of acid treatment, the film thickness first grew and then decreased. To test this, we measured the thickness change with acid treating time. As seen in Figure 3B, film thickness changed from 634.2 ± 35.9 nm up to 866.15 ± 48.0 nm at the first 6 min. However, film thickness decreased to 730.2 ± 64.4 nm at 8 min, 727.0 ± 25.0 nm at 10 min, and 723.7 ± 23.1 nm at 15 min. The detailed information was measured further through SEM (Figure S5, Supporting Information). The periodic stratified porous structure was invisible after acid treatment for 2 min, and became more and more distinct after prolong the treating time. While, after acid treatment for 8 min, the structure became vague, and with treating time increasing, it showed a much more indistinct state. Such phenomena might arise because increasing the acid treatment time, the porous layers could be thicker until the collapse happened when the edge of the pores became too thin to support the solid layers. In short, through control the acid treatment time, we can control the layer spacing of the stratified porous structure, leading to diversified structural color.

Given that the dynamic property of non‐crosslinked polyelectrolyte films in acid solution and 100% RH environment,^[8a,9c,17]^ the periodic stratified porous (PEI/PAA‐N_3_)_5_ film retained the possibility of further structural controlment because of the presence of unconstrained polyelectrolytes in the non‐crosslinked layers. To test this, the (PEI/PAA‐N_3_)_5_ film with periodic stratified porous structures was exposed to 100% RH environment for 12 h. The film thickness decreased from 809.5 ± 22.6 to 654.3 ± 8.4 nm as shown in Figure 3C, corresponding to the disappearance of the porous structure as expected (Figure 3D). After that, repeating acid treatment and exposure of 100% RH environment for three times, we generated the stratified structures and solid‐state in the (PEI/PAA‐N_3_)_5_ film reversibly. Correspondingly, the film thickness changed regularly (Figure 3C). The procedures could be repeated three times. In a word, the structure of the (PEI/PAA‐N_3_)_5_ film could be tuned dynamically and reversibly.

### 2.5 Structural Color Patterning of (PEI/PAA‐N_3_)_5_ Film

**Figure 4:**
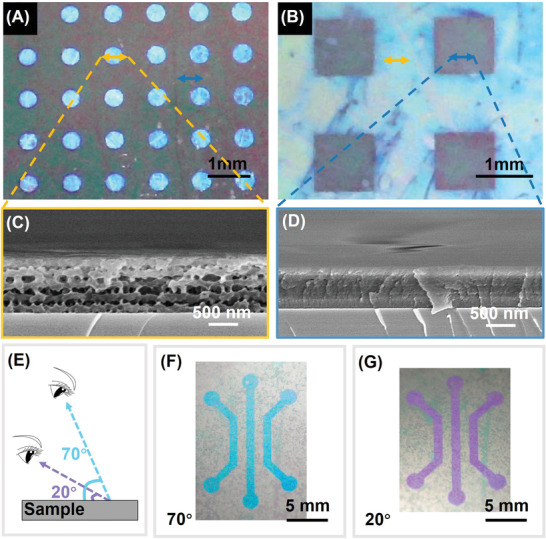
The photographs of structural color patterns. A) Circular matrix with stratified porous structure in circular regions. B) Square matrix with stratified porous structure around the squares. C) The cross‐sectional images of stratified porous structures corresponding to the regions where the yellow lines locate. D) The cross‐sectional images of the solid film corresponding to the regions where the blue lines locate. E) The schematic of capturing the structural color patterns at different angles (70° and 20°). The photographs of pattern in a (PEI/PAA‐N_3_)_5_ film were captured at F) 70° and G) 20°.

Generating structural color patterns could serve as an environmentally friendly and effective method for various applications such as ink‐free printing,^[12a,25]^ detection,^[^ ^24^ ^]^ sensors,^[^ ^26^ ^]^ etc. By taking advantage of the different pore‐formation properties under different irradiation conditions (Figure 1D and Figure S2: Supporting Information), various structural color patterns composed of stratified porous structural regions and solid regions can be developed. To display this, we prepared a bright blue dot matrix as shown in a (PEI/PAA‐N_3_)_5_ film (**Figure** **4A**). The bright blue regions corresponded to the stratified porous structural regions (Figure 4C) while the gray regions kept solid‐state (Figure 4D). Through changing the pre‐designed photo mask, we adjusted the distribution of stratified porous and solid regions and subsequently got a different pattern. As shown in Figure 4B, a square matrix was fabricated. Similarly, the bright blue region was stratified porous while the gray square regions were solid. That is, combining the standing‐wave optics and regional irradiation under the protection of a photo mask to control the crosslinking of (PEI/PAA‐N_3_)_5_ film laterally and vertically, we successfully obtained different structural color patterns. Last but not least, in this study, the stratified porous structural (PEI/PAA‐N_3_)_5_ films displayed different colors when being observed at different angles (Figure S6, Supporting Information), which led to the development of a color‐changeable pattern. The structural color logo of Zhejiang University was displayed on a (PEI/PAA‐N_3_)_5_ film (Figure 4F). A bright blue logo was captured at 70° (the included angle between sightline and horizon line) when a purple logo was observed at 20° (Figure 4E–G). The color‐changeable pattern can be ascribed to the characteristics from optical interference,^[^ ^27^ ^]^ and the character of changeable color endows the polyelectrolyte film with broader application potential.

## 3 Conclusions

In this study, we established a simple approach to generate the periodic stratified porous structures in dynamic polyelectrolyte film through periodic crosslinking based on standing‐wave optics. We first prepared a photo‐cross‐linkable (PEI/PAA‐N_3_)_5_ film by grafting azido groups into polyanion. Utilizing standing‐wave optics, periodic crosslinking was introduced into the film. The pores only formed in the non‐crosslinked layers. In this way, we constructed periodic stratified porous structures into the (PEI/PAA‐N_3_)_5_ film. The intrinsic dynamics of polyelectrolyte film endowed its structure controlment with adjustability and reversibility. Further, we prepared a periodic stratified porous pattern in (PEI/PAA‐N_3_)_5_ film with changeable structural color. This study provides an effective method of introducing periodic structure into polyelectrolyte film, which widens the application potential of the polymeric film.

## 4 Experimental Section

### Materials

Branched PEI (*M*_w_ 25 000), PAA (35 wt%, *M*_w_ 100 000), and 4‐azidoaniline hydrochloride were purchased from Sigma‐Aldrich (Shanghai, China). 1‐Ethyl‐3‐(3‐(dimethylamino)propyl)‐carbodiimide hydrochloride (EDC), *N*‐hydroxysulfosuccinimide sodium salt (NHSS) were from Aladdin (Shanghai, China). Deionized water used in all experiments was obtained through a Milli‐Q water purification system (Millipore, Billerica, America). The pH of various aqueous solutions was adjusted by 1.0 m HCl or 1.0 m NaOH as needed.

### Synthesis of PAA‐N_3_

The photo‐responsive polyanion (PAA‐N_3_) was synthesized through the amidation between carboxyl groups of PAA and amino groups of 4‐azidoaniline hydrochloride. Briefly, 1.42 g PAA and 0.07 g 4‐azidoaniline hydrochloride were dissolved into 50 mL ultrapure water after stirring for 30 min. Adjusting the pH of the above aqueous solution at 7. Adding 0.24 g EDC and 0.48 g NHSS into the solution. There was a sustained reaction for 24 h at 4 °C. After that, the mixture was dialyzed against ultrapure water for 3 days, and the final solution was freeze‐dried in a vacuum (1 Pa) at −50 °C for 3 days to get a flocculent white solid. The grafting ratio of PAA‐N_3_ was calculated according to the integrals of the three hydrogen atoms on the polymeric bone and the four hydrogen atoms on the aromatic ring in the ^1^H‐NMR spectrum.

### PEI/PAA‐N_3_ Film Buildup

Substrates (glass, quartz, and silicon wafers) were immersed into a fresh piranha solution (30% H_2_O_2_/98% H_2_SO_4_ = 3/7 v/v) for 30 min, rinsed with ultrapure water more than three times, and then blow‐dried by nitrogen (N_2_). A PEI/PAA‐N_3_ film was prepared by first dipping substrate into PEI aqueous solution (1 mg mL^−1^, pH 9.0) for 15 min, flushing with ultrapure water thoroughly, and blow‐drying by N_2_. The substrate was dipped into PAA‐N_3_ aqueous solution (3 mg mL^−1^, pH 4.0) for another 15 min, followed by the same rinsing and drying procedures. These steps were repeated until the needed number of bilayers was obtained. The film will be expressed as (PEI/PAA‐N_3_)*_n_*, where *n* is the number of the bilayer. Exposing the films into relative saturated humidity (100% RH) environment for 12 h to obtain smoother and more solid samples. The pre‐treated PEI/PAA‐N_3_ films were regarded as the as‐prepared films for the following experiments and characterizations.

### Crosslinking of PEI/PAA‐N_3_ Film

The whole crosslinking of the (PEI/PAA‐N_3_)_5_ films was achieved after being exposed to UV irradiation in crosslinking oven No.1 (50 mW cm^−2^, Uvitron Intelli‐Ray 400, broad wavelength range) for the 60 s. Stratified crosslinking of PEI/PAA‐N_3_ films should be more cautious. Specifically, the (PEI/PAA‐N_3_)_5_ films deposited on silicon wafers were illuminated directly in crosslinking oven No.2 (FUWO, LED light resources, 365 nm) while the films deposited on transparent substrates should set closely onto a clean silicon wafer before illumination. The silicon wafer here provided the plane of reflection for the formation of a standing‐wave. The samples were then exposed to UV irradiation (51.9 mW cm^−2^) for 60 s to achieve the stratified crosslinking. It is worth mentioning that standing‐wave optics can be developed effectively when a beam with a single wavelength illuminates onto a flat and reflected surface.

### Creation and Erasure of Microporous Structure

Porous structures within (PEI/PAA‐N_3_)_5_ films were created by immersing the sample into a bath of hydrochloric acid solution (pH 2.3) for different time (2, 4, 6, 8, 10, and 15 min). After that, the film was frozen at −80 °C for 30 min suddenly and then freeze‐dried to reserve the porous structure. It is worth mentioning that the whole crosslinked (PEI/PAA‐N_3_)_5_ films cannot be porous. As for stratified crosslinked films, crosslinked regions did not form micropores, while the non‐crosslinked regions can do it. The erasure of the microporous structures was enabled by exposing the porous (PEI/PAA‐N_3_)_5_ films into 100% RH environment for 12 h.

### Fabrication of the Microporous Patterns

Microporous patterns in (PEI/PAA‐N_3_)_5_ films can be classified into two kinds of patterns: 1) the combination of solid regions and multilayered porous regions; 2) the combination of whole porous regions and multilayered porous regions. As for the first case, the designed pattern was written into (PEI/PAA‐N_3_)_5_ films under the protection of photo mask after UV irradiation for 60 s (in crosslinking oven No.1). The transparent and opaque regions in photo mask corresponded to whole‐crosslinked and non‐crosslinked regions respectively. Then the film was illuminated for another 60 s without the photomask in crosslinking oven No.2. The original non‐crosslinked regions were induced the stratified crosslinking. Finally, immersing the film into a bath of hydrochloric acid solution (pH 2.3) for 6 min and freeze‐drying it to obtain the pattern combined solid regions with multilayered porous regions. For the second condition, the designed pattern was written into (PEI/PAA‐N_3_)_5_ films under the protection of photo mask after UV irradiation for 60 s in crosslinking oven No.2. Herein, the transparent and opaque regions in the photo mask corresponded to the stratified‐crosslinked and non‐crosslinked regions in (PEI/PAA‐N_3_)_5_ films. Then the film was directly immersed into an acid solution (pH 2.3) for 6 min. After freeze‐dried, the pattern combined whole porous regions and multilayered porous regions was prepared.

### Characteristics

The chemical structure of PAA‐N_3_ was analyzed by ^1^H‐NMR (DMX‐500, Bruker, Switzerland). The thickness changes in films were measured by a stylus profiler (DekTak‐XT, Bruker, Germany). A UV−vis spectrophotometer (UV‐2550, Shimadzu, Japan) was used to record the absorbance spectra of PEI/PAA‐N_3_ films. The reflectance spectra of (PEI/PAA‐N_3_)_5_ films were characterized by another UV−vis spectrophotometer (UV‐3600, Shimadzu, Japan) with an external reflection testing chamber. SEM (Hitachi S4800, Japan, and Hitachi SU8010, Japan) was performed to explore the structure of the films. Young's modulus of the (PEI/PAA‐N_3_)_5_ film was obtained from nanoindenter (Piuma) with a 44.6 N m^−1^ probe. Dataviewer software (Piuma) was used to calculate the Young's modulus. The photographs were captured by a digital camera (ILCE‐A6000, Sony).

### Statistical Analysis

All the experimental data are expressed as mean ± SD. An error bar of one standard deviation is added in the data wherever applicable. Comparative analysis of differences of Young's modulus was performed using the two‐tailed Student's *t*‐test (Excel software, Microsoft). Significance was considered as *p < 0.05, **p < 0.01, and ***p < 0.001. All the other data were analyzed and plotted with OriginPro 2018 (OriginLab Corp.).

## Conflict of Interest

The authors declare no conflict of interest.

## Supporting information

Supporting Information

## Data Availability

Research data are not shared.

## References

[1] a) I. Tokarev , S. Minko , Adv. Mater. 2010, 22, 3446; 20473983 10.1002/adma.201000165

[2] Y. Qi , W. Niu , S. Zhang , S. Wu , L. Chu , W. Ma , B. Tang , Adv. Funct. Mater. 2019, 29, 1906799.

[3] J. Kang , J. Kim , S. Lee , S. Wi , C. Kim , S. Hyun , S. Nam , Y. Park , B. Park , Adv. Energy Mater. 2017, 7, 1700814.

[4] C. Chen , L. Hu , Adv. Mater. 2021, 2002890. 10.1002/adma.202002890 33108027

[5] A. Finnemore , P. Cunha , T. Shean , S. Vignolini , S. Guldin , M. Oyen , U. Steiner , Nat. Commun. 2012, 3, 966. 22828626 10.1038/ncomms1970

[6] B. Wang , T. N. Sullivan , A. Pissarenko , A. Zaheri , H. D. Espinosa , M. A. Meyers , Adv. Mater. 2019, 31, 1804574. 10.1002/adma.201804574 30450716

[7] a) J. Borges , J. F. Mano , Chem. Rev. 2014, 114, 8883; 25138984 10.1021/cr400531v

[8] a) J. D. Mendelsohn , C. J. Barrett , V. V. Chan , A. J. Pal , A. M. Mayes , M. F. Rubner , Langmuir 2000, 16, 5017;

[9] a) X.‐C. Chen , K.‐F. Ren , J.‐Y. Chen , J. Wang , H. Zhang , J. Ji , Phys. Chem. Chem. Phys. 2016, 18, 31168; 27819085 10.1039/c6cp05419f

[10] X.‐C. Chen , K.‐F. Ren , W.‐X. Lei , J.‐H. Zhang , M. C. Martins , M. A. Barbosa , J. Ji , ACS Appl. Mater. Interfaces 2016, 8, 4309. 26844588 10.1021/acsami.5b11602

[11] W.‐P. Huang , X.‐C. Chen , M. Hu , J. Wang , H. L. Qian , D. F. Hu , R. L. Dong , S. Y. Xu , K. F. Ren , J. Ji , ACS Appl. Mater. Interfaces 2020, 12, 42081. 32937689 10.1021/acsami.0c09580

[12] a) M. M. Ito , A. H. Gibbons , D. Qin , D. Yamamoto , H. Jiang , D. Yamaguchi , K. Tanaka , E. Sivaniah , Nature 2019, 570, 363; 31217598 10.1038/s41586-019-1299-8

[13] G. Wu , F. Shi , Z. Wang , Z. Liu , X. Zhang , Langmuir 2009, 25, 2949. 19437767 10.1021/la804261f

[14] a) X. C. Chen , W. P. Huang , M. Hu , K. F. Ren , J. Ji , Small 2019, 15, 1804867; 10.1002/smll.201804867 30677229

[15] Q. An , T. Huang , F. Shi , Chem. Soc. Rev. 2018, 47, 5061. 29767189 10.1039/c7cs00406k

[16] a) C. Picart , J. Mutterer , L. Richert , Y. Luo , G. D. Prestwich , P. Schaaf , J. C. Voegel , P. Lavalle , Proc. Natl. Acad. Sci. USA 2002, 99, 12531; 12237412 10.1073/pnas.202486099 PMC130494

[17] H. M. Fares , J. B. Schlenoff , J. Am. Chem. Soc. 2017, 139, 14656. 28981268 10.1021/jacs.7b07905

[18] M. Hu , H. Chang , H. Zhang , J. Wang , W. X. Lei , B. C. Li , K. F. Ren , J. Ji , Adv. Healthcare Mater. 2017, 6, 1601410. 10.1002/adhm.201601410 28474486

[19] H. H. Hariri , A. M. Lehaf , J. B. Schlenoff , Macromolecules 2012, 45, 9364.

[20] M. Qin , M. Sun , M. Hua , X. He , Curr. Opin. Solid State Mater. Sci. 2019, 23, 13.

[21] H. S. Lim , J. H. Lee , J. J. Walish , E. L. Thomas , ACS Nano 2012, 6, 8933. 23020142 10.1021/nn302949n

[22] Y. Kang , J. J. Walish , T. Gorishnyy , E. L. Thomas , Nat. Mater. 2007, 6, 957. 17952084 10.1038/nmat2032

[23] J. J. Walish , Y. Kang , R. A. Mickiewicz , E. L. Thomas , Adv. Mater. 2009, 21, 3078.

[24] M. Sun , R. Bai , X. Yang , J. Song , M. Qin , Z. Suo , X. He , Adv. Mater. 2018, 30, 1804916. 10.1002/adma.201804916 30252962

[25] a) V. V. Doan , M. J. Sailor , Science 1992, 256, 1791; 17743033 10.1126/science.256.5065.1791

[26] a) J. H. Lee , B. Fan , T. D. Samdin , D. A. Monteiro , M. S. Desai , O. Scheideler , H. E. Jin , S. Kim , S. W. Lee , ACS Nano 2017, 11, 3632; 28355060 10.1021/acsnano.6b07942

[27] S. Wu , T. Liu , B. Tang , L. Li , S. Zhang , ACS Appl. Mater. Interfaces 2019, 11, 10171. 30757893 10.1021/acsami.8b21092

