# Supplementary material for: Periodic Stratified Porous Structures in Dynamic Polyelectrolyte Films Through Standing‐Wave Optical Crosslinking for Structural Color
Source: Adv Sci (Weinh). 2021 May 27;8(15):2100402. doi: 10.1002/advs.202100402 (PMC8336486; doi:10.1002/advs.202100402)
Supplement: Supplementary file 1 — Supporting Information [file ADVS-8-2100402-s001.pdf]

WILEY-VCH

© 2021 Wiley-VCH GmbH

ADVANCED SCIENCE
Open Access

Supporting Information

for *Adv. Sci.*, DOI: 10.1002/advs.202100402

Periodic Stratified Porous Structures in Dynamic Polyelectrolyte Films Through Standing-wave Optical Crosslinking for Structural Color

Wei-Pin Huang, Hong-Lin Qian, Jing Wang, Ke-Feng Ren,, and Jian Ji*

Supporting Information

Periodic stratified porous structures in dynamic polyelectrolyte films through standing-wave optical crosslinking for structural color

Wei-Pin Huang[1], *Hong-Lin Qian*[1], *Jing Wang*[1], *Ke-Feng Ren*[1,2,*], *and Jian Ji*[1,2]

[1]MOE Key Laboratory of Macromolecule Synthesis and Functionalization, Department of Polymer Science and Engineering, Zhejiang University, Hangzhou 310027, China.
[2] Key Laboratory of Cardiovascular Intervention and Regenerative Medicine of Zhejiang Province, Department of Cardiology, Sir Run Run Shaw Hospital, Zhejiang University, Hangzhou 310016, China.

*E-mail：renkf@zju.edu.cn

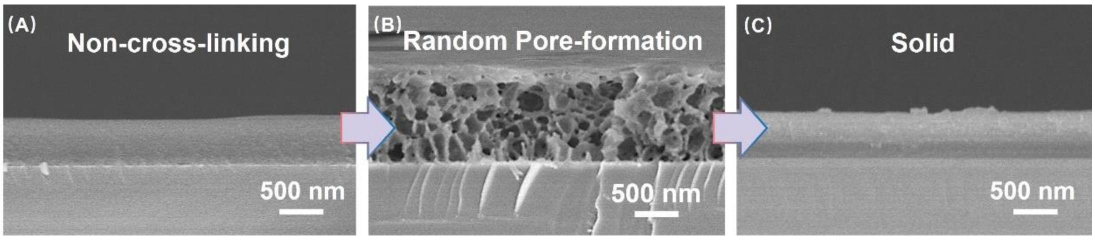

Figure S1. The cross-sectional SEM images of the non-cross-linked (PEI/PAA-N_3)$_5$ film at initial state (A) and after pore-formation (B) in a bath of HCl aqueous solution (pH 2.3) for 6 min. (C) The cross-sectional SEM image of porous (PEI/PAA-N_3)$_5$ film after 100% RH environment for 12 h.

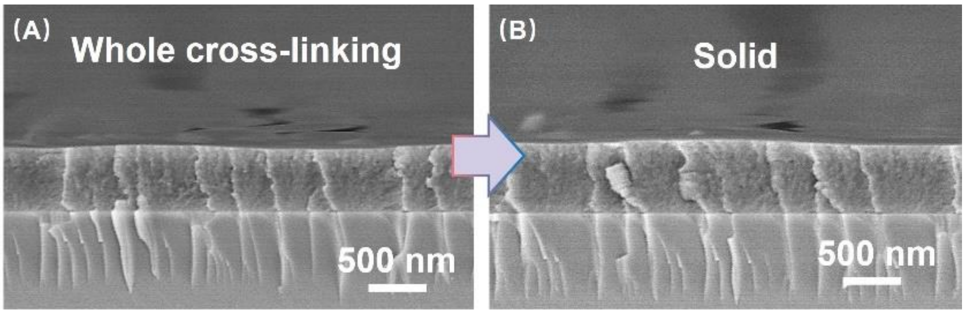

Figure S2. The cross-sectional SEM images of the whole cross-linked (PEI/PAA-N_3)$_5$ film at initial state (A) and after pore-formation (B) in a bath of HCl aqueous solution (pH 2.3) for 6 min.

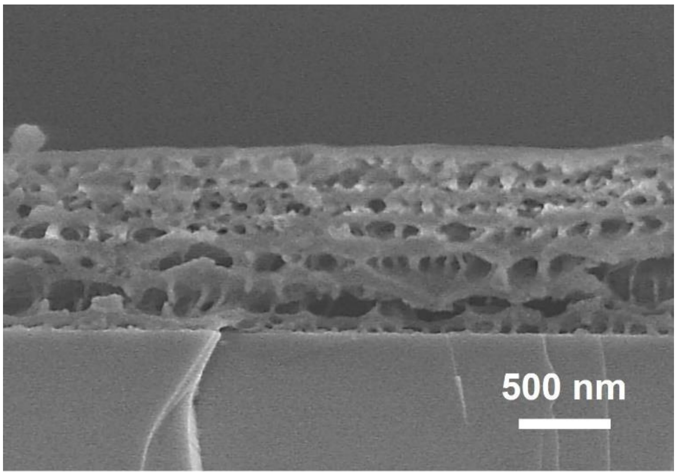

Figure S3. The cross-sectional SEM image of the periodic stratified porous (PEI/PAA-N_3)$_6$ film.

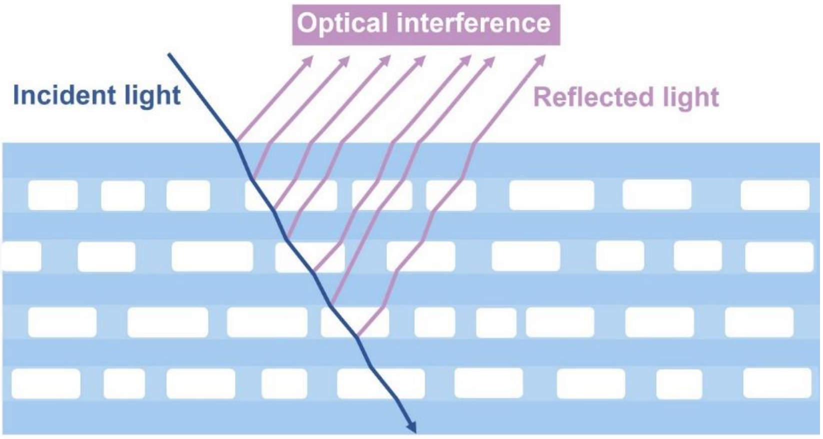

Figure S4. The schematic of the generation of structural color in stratified porous $(PEI/PAA\text{-}N_3)_5$ films.

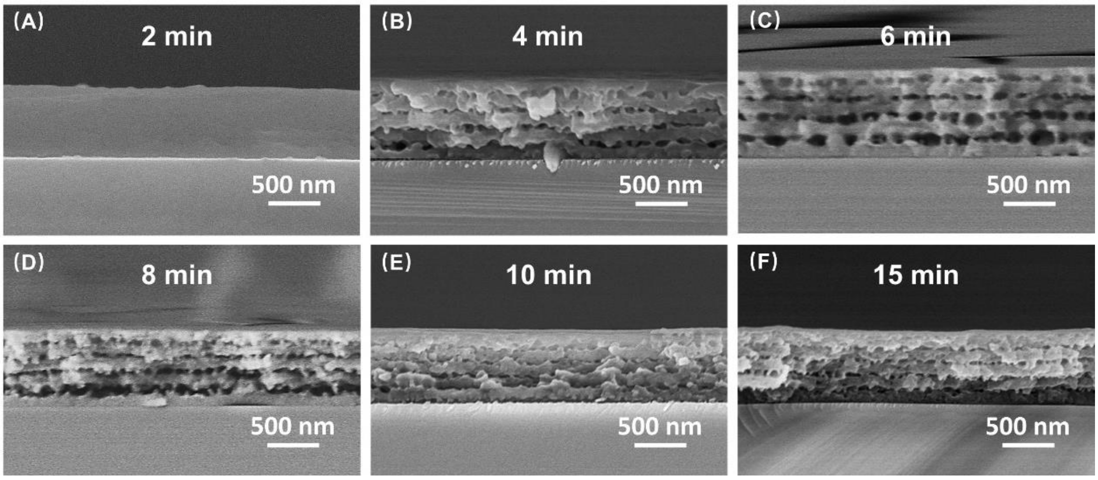

Figure S5. The cross-sectional SEM images of the (PEI/PAA-N_3)$_5$ films after acid treatment (HCl aqueous solution, pH 2.3) for different time (2, 4, 6, 8, 10, 15 min).

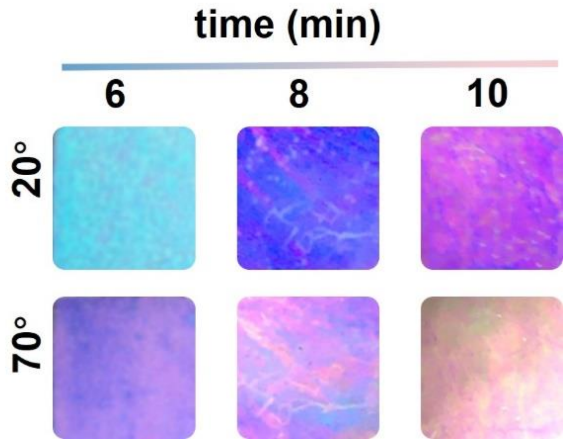

Figure S6. The different colors when being observed from different perspectives for the $(PEI/PAA-N_3)_5$ films after acid treatment for different time (6, 8, 10min).
